# The effects of fucodian on senescence are controlled by the p16^INK4a^-pRb and p14^Arf^-p53 pathways in hepatocellular carcinoma and hepatic cell lines

**DOI:** 10.3892/ijo.2014.2426

**Published:** 2014-05-08

**Authors:** EUN-YOUNG MIN, IN-HYE KIM, JUNGIM LEE, EUN-YOUNG KIM, YOUN-HEE CHOI, TAEK-JEONG NAM

**Affiliations:** Institute of Fisheries Sciences, Pukyong National University, Ilgwang-ro, Ilgwang-myeon, Gijang-gun, Busan 619-911, Republic of Korea

**Keywords:** fucoidan, anti-tumor, anti-senescence, p16^INK4a^-Rb, p14^Arf^-p53, α_2_M

## Abstract

Fucoidan is known to have various pharmacological effects, including antitumor activity. Although it has potential as a therapeutic agent for cancer cells, the anti-senescence effects and detailed mechanism of action remain poorly understood in normal hepatic cells. We investigated the anticancer functions of fucoidan using HepG2 cells as well as the mechanisms mediating the anti-senescent actions in Chang liver cells. Fucoidan effectively inhibited HepG2 cell viability and induced apoptosis. Also, fucoidan-induced G_1_ phase arrest was caused by the activity of the p16^INK4a^-Rb and p14^Arf^-p53 pathways. Furthermore, upregulation of p16^INK4a^ was critical to the antitumor activity of HepG2 cells treated with fucoidan and was correlated with inhibition of Cdk4 and pRb and upregulation of p21 expression. Our results suggest that fucoidan upregulates *INK4a* locus genes to induce apoptosis through p38 MAPK in HepG2 cells. Moreover, it prevents cellular senescence of Chang-L cells, by decreasing p14^Arf^ expression as cells enter quiescence, with the reduction of p16^INK4a^. Fucoidan treatment also downregulated the expression of α_2_M. In conclusion, fucoidan can be considered a potential therapeutic agent against liver cancer that does not cause senescence in normal hepatic cells. Thus, it may be possible to use fucoidan therapeutically in both tumor suppression and aging.

## Introduction

Fucoidan is a polysaccharide from the cell wall of brown seaweed containing a substantial percentage of L-fucose and sulfate ester groups (1). It has numerous pharmacological properties as an anti-coagulant, anti-tumor, anti-inflammatory and anti-oxidant agent (2–5). In particular, its anti-tumor activity has recently attracted considerable attention (6,7) as a potential therapeutic agent for cancer. However, the anti-senescence effects and detailed mechanism of action remains poorly understood in normal hepatic cells.

Treatment options for liver cancer focus primarily on chemotherapy (8). While these chemotherapeutic regimens are well established, the major drawback remains their limited specificity for the tumor site. Chemotherapeutic drugs, such as cisplatin, induce senescence by enhancing the activity of the tumor suppressor p16^INK4a^ in cancer and normal cells, which results in increased toxicity to normal cells, which require balanced expression of p16^INK4a^ for growth and differentiation to maintain cell homeostasis. Thus, cancer cell-specific expression of p16^INK4a^ would be a valuable therapeutic strategy for cancer treatment (9).

**Figure 1.**
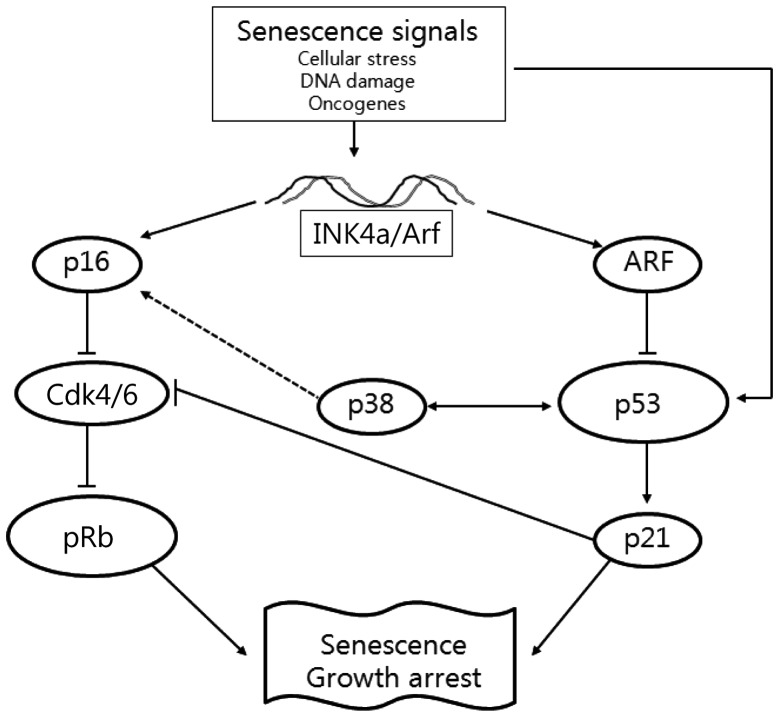
The p16^INK4a^-pRb and p14^Arf^-p53 pathways mediate the hyperproliferative stress response in cancer. Hyperproliferative signals including cellular stress, DNA damage and oncogenes lead to increased levels of p16^INK4a^ and p14^Arf^. p16^INK4a^ inhibits Cdk4/6 activity, inducing Rb phosphorylation, which leads to cell cycle arrest. p14^Arf^ inhibits MDM2-mediated degradation of p53, which activates p21, a Cdk inhibitor. Through an unknown mechanism, active p38 MAPK also induces the expression of p16^INK4a^ and p14^Arf^, which together with the p53-p21 cascade, causes premature senescence that serves as a tumor-suppressing defense mechanism *in vitro* and *in vivo*.

Cellular senescence, leading to cell death through the prevention of regular cell renewal, is associated with the upregulation of p16^INK4a^ in most mammalian tissues (10). This process requires activation of several signaling pathways, including phosphorylated retinoblastoma (pRb) and p14^Arf^-p53 (11). Phosphorylation of the Rb protein results in increased p16^INK4a^ expression to inhibit cyclin-dependent kinase (Cdk) 4/6. This leads to increased levels of hypophosphorylated Rb that decrease p16^INK4a^ expression (12). Although there is a feedback loop between p16^INK4a^ and Rb, p16^INK4a^ expression does not change appreciably during the cell cycle to correlate with the activation status of Rb (13). However, increased expression of p16^INK4a^ leads to senescence and cancer cells inactivate p16^INK4a^ by homozygous deletion or hypermethylation to overcome its effects. The tumor suppressor p14^Arf^ (p19^Arf^ in mouse cells) has emerged as an interesting candidate linking transformation and senescence responses. Arf is the second protein, in addition to p16, expressed from the *INK4a*/*Arf* locus, but bears no homology to p16 or any other cyclin-dependent kinase inhibitors (CKIs) (14). Arf neutralizes the ability of MDM2 to promote p53 degradation, leading to the stabilization and accumulation of p53 (15). Increased expression of Arf also causes growth arrest, one of the hallmarks of premature senescence (16). Thus, the tumor suppressor proteins of the *INK4a*/*Arf* locus function in distinct anticancer pathways as p16^INK4a^ directly regulates pRb, while p14^Arf^ directly regulates p53 and indirectly regulates pRb (Fig. 1). Inactivation of the p53 and pRb pathways through a variety of mechanisms occurs in the majority of, if not all, human cancers. Although these pathways play important roles in differentiation, development and DNA repair, the *INK4a*/*Arf* locus responds largely to aberrant growth or oncogenic stress. Therefore, the *INK4a*/*Arf* locus appears to function as a dual-pronged brake to malignant growth, which engages two potent anti-proliferative pathways represented by p16^INK4a^-pRb and p14^Arf^-p53 signaling (17).

The p38 mitogen-activated protein kinase (MAPK) pathway regulates cellular processes that directly contribute to tumor suppression, including oncogene-induced senescence and replicative senescence (18), as well as proliferation and tumorigenesis (19). Senescence is also accompanied by markers associated with replicative exhaustion of normal cells, such as senescence-associated β-galactosidase (SA-β-gal) activity and the induction of p21, p16^INK4a^ and/or p14^Arf^ (16,20). A previous study suggested that expression of α-2-macroglobulin (α_2_M) can be used as a biomarker of aging in cultured human fibroblasts, can be measured easily by reverse-transcriptase polymerase chain reaction (RT-PCR) with a limited sample, and is a more suitable biomarker candidate of aging then the well-known senescence-associated genes such as p16^INK4a^ (21). In this study, we determined the expression of α_2_M as a biomarker of cellular senescence to assess the anti-senescence effects of fucoidan in normal human liver cells.

## Materials and methods

### Fucoidan

Commercially available fucoidan purified form *F. vesiculosus* (F5631) was purchased from Sigma-Aldrich, Inc. (St. Louis, MO, USA).

### Cell culture

The human hepatocellular carcinoma cell line (HepG2; HB-8065) and the human normal liver cell line (Chang-L; CCL-13) were obtained from the American Type Culture Collection (ATCC, GA, USA). Cells were cultured in MEM medium supplemented with 10% fetal bovine serum, penicillin (100 U/ml) and streptomycin (100 *μ*g/ml) at 37°C in a humidified 5% CO_2_ incubator.

### Cell viability assay

Cell viability was determined by the Cyto^™^ cell viability assay kit (LPS solution, Daejeon, Korea). Cells were seeded at a density of 1×10^4^ cells/well in a 96-well plate. After 24 h, the cells were treated with the phosphate-buffered saline (PBS) vehicle or 100, 250 and 500 *μ*g/ml fucoidan. The Cyto solution was added to each well and incubated for 4 h. The formazan product was estimated by measuring absorbance at 450 nm in a microplate reader (BioTek Instruments, Inc., Winooski, VT, USA). The viability of fucoidan-treated cells was expressed as a percentage of vehicle-treated control cells considered 100% viable.

### Cell cycle analysis

Cells were seeded at a density of 1×10^4^ cells/well and treated with various concentrations of fucoidan for 24 h. Control and treated cells were harvested, washed in cold PBS, fixed in 70% ethanol and stored at 4°C. The resulting cells were stained with 200 *μ*l of Muse^™^ cell cycle reagent at room temperature for 30 min in the dark before analysis. DNA content was assessed by Muse^™^ cell analyzer (EMD Millipore Co., CA, USA).

### Apoptosis analysis

The Muse Annexin V and Dead cell kit (EMD Millipore Co., MA, USA) was used for the apoptosis assay. HepG2 cells and Chang-L cells plated at a density of 1×10^6^ cells/well were treated with varying concentrations of fucoidan for 24 h. Cells were harvested by trypsinization, washed twice with PBS, and re-suspended in Annexin V and 7-aminoactinomycin D (7-AAD) for 20 min at room temperature in the dark. The cells were evaluated immediately by Muse cell analyzer. The percentage of apoptotic cells was assessed using the Muse^™^ software.

### Western blotting

Samples were analyzed by western blotting, as described previously (20). Whole-cell lysates were prepared by lysing cell pellets in a NETN lysis buffer [0.5% Nonidet P-40, 1 mM EDTA, 50 mM Tris (pH 7.4), 12 mM NaCl, 1 mM DTT, 10 mM NaF, 2 mM Na_3_VO_4_, 1 mM PMSF]. Samples (50 *μ*g) were resolved by SDS-PAGE and transferred to Immobilion-P transfer membranes (Millipore Co., MA USA). The primary antibodies used included monoclonal anti-p16^INK4a^, polyclonal anti-Cdk4 and -Cdk6, polyclonal anti-p21, polyclonal anti-p38, monoclonal anti-p-p38, polyclonal anti-p53 and polyclonal anti-pRb (1:1,000, Santa Cruz Biotechnology, Inc., TX, USA). Membranes were washed and incubated with the corresponding HRP-conjugated secondary antibody at 1:10,000. Bound secondary antibody was detected using a chemiluminescence substrate (Advansta, Menlo Park, CA, USA) and visualized on GeneSys imaging system (SynGene Synoptics, Ltd., London, UK).

### Real-time PCR

Cells were harvested 24 h after treatment with PBS, 100, 250 or 500 *μ*g/ml fucoidan. Total-RNA was extracted from HepG2 and Chang-L cells using the QIAzol lysis reagent (Qiagen Sciences, Inc., Germantown, MD, USA). RNA quality was evaluated by measuring absorbance at 260 and 280 nm to calculate the concentration and to assess the purity of RNA, respectively. Agarose electrophoresis was used to detect RNA purity and integrity.

The GoScript^™^ Reverse Transcription System (Promega Corp., Madison, WI, USA) was used to prepare cDNA according to the manufacturer’s instructions; the samples were stored at −20°C. The quality of cDNA was assessed by amplifying an internal reference gene, glyceraldehyde 3-phosphate dehydrogenase (GAPDH), by PCR and the results were confirmed by 2% agarose gel electrophoresis. The products were examined by computerized gel imaging system (Bio-Rad, Hercules, CA, USA).

Quantitative PCR was conducted in 20 *μ*l reactions containing QuantiMix SYBR kit (PhilKorea Technology, Inc., Daejeon, Korea) using the Illumina Eco^™^ real-time PCR system (Illumina, Inc., Hayward, CA, USA). The oligonucleotide primers for p16^INK4a^, p14^Arf^, p21, p53, p38, α_2_M and GAPDH are shown in Table I. Reaction mixtures were incubated for an initial denaturation at 95°C for 10 min followed by 40 cycles of 95°C for 15 sec, 55°C for 15 sec and 72°C for 15 sec. For each sample, the expression level of each mRNA was quantified as the cycle threshold difference (ΔC_t_) to GAPDH mRNA. All reactions were performed in triplicate and repeated with two independent experiments.

### Statistical analysis

SPSS software (Chicago, IL, USA) was used to perform the statistical analysis. For comparisons for more than two groups, data were analyzed by one-way analysis of variance (ANOVA), followed by Duncan’s test for multiple comparisons. For all tests, P<0.05 was considered to indicate significance.

## Results

### Effects of fucoidan on cell viability

**Figure 2.**
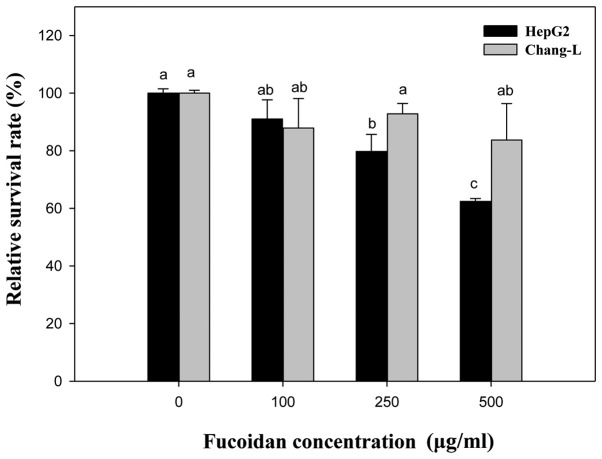
The effects of fucoidan on cell viability. HepG2 and Chang-L cells were incubated with increasing concentrations of fucoidan for 24 h. Concentration-dependent changes in cell viability were determined by the Cyto cell viability assay kit. Data represent means ± standard deviation in trip-licate. Different superscripts represent significantly different results (P<0.05).

To verify the effects of fucoidan on cell viability, cells were treated with fucoidan at the concentrations indicated for 24 h (Fig. 2). Compared to the untreated controls, Chang-L cells exhibited no cytotoxicity at concentrations between 0 and 500 *μ*g/ml. In contrast, proliferation of HepG2 cells was dose-dependently inhibited by fucoidan treatment (250 *μ*g/ml, 79.75±9.94% inhibition; 500 *μ*g/ml, 62.43±1.0% inhibition). Thus, the ability of fucoidan to inhibit proliferation was significantly different between HepG2 and Chang-L cells.

### Effects of fucoidan on apoptosis

**Figure 3.**
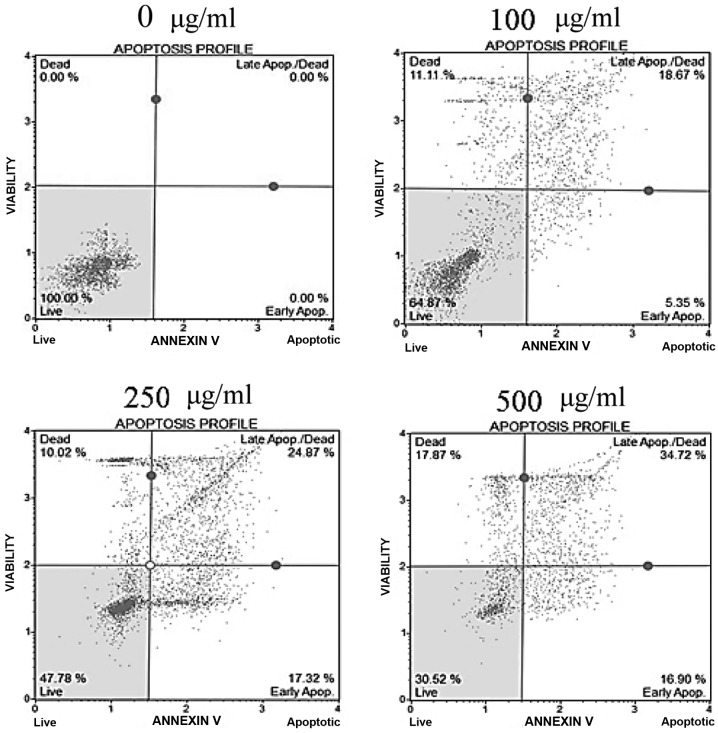
Fucoidan treatment induces dose-dependent apoptosis in HepG2 cells. After treatment with increasing concentrations of fucoidan for 24 h, apoptosis was assessed using a cell analyzer. ‘Early Apop’ indicates the percentage of early apoptotic cells (Annexin V-stained cells) and ‘Late Apop/Dead’ indicates the percentage of late apoptotic cells (Annexin V + 7-AAD-stained cells).

To determine whether the cytotoxicity of fucoidan was caused by apoptosis, Annexin V/7-AAD double-staining was performed. In fucoidan-treated HepG2 cells, the percentage of the early apoptotic cells, as well as the total percentage of Annexin V-positive cells indicating late apoptotic cells, was significantly increased in a dose-dependent manner (Fig. 3). In Chang-L cells, the percentage of apoptotic cells did not differ between fucoidan-treated groups and controls (data not shown). These results indicate that fucoidan had a strong antitumor effect on hepatocellular carcinoma cells and is a potent apoptosis-inducing agent.

### Effects of fucoidan on the cell cycle

To determine whether fucoidan affected the cell cycles of HepG2 and Chang-L cells, we performed cell analysis 24 h after fucoidan treatment. Treatment with 500 *μ*g/ml fucoidan led to a significant decrease in the production of S and G_2_/M phases and G_0_/G_1_ phase arrest in HepG2 cells (43.12% in W/O, 77.78% in fucoidan-treated samples; P<0.05). Treatment at concentrations between 0 and 500 *μ*g/ml did not significantly change the cell cycles of Chang-L cells (Table II). These results suggest that the anti-proliferative effect of fucoidan on HepG2 cells can be attributed to a blocking of the G_0_/G_1_ phase of the cell cycle.

### Expression of p16^INK4a^-pRb pathway-related proteins in fucoidan-treated cells

To evaluate the mechanism underlying the tumor-suppressing activity of fucoidan, we examined the expressions of p16^INK4a^, Cdk4/6 and pRb by western blotting in HepG2 cells after treatment with fucoidan for 24 h. We further explored the mechanism of this antitumor action by evaluating mRNA expression by real-time PCR.

**Figure 4.**
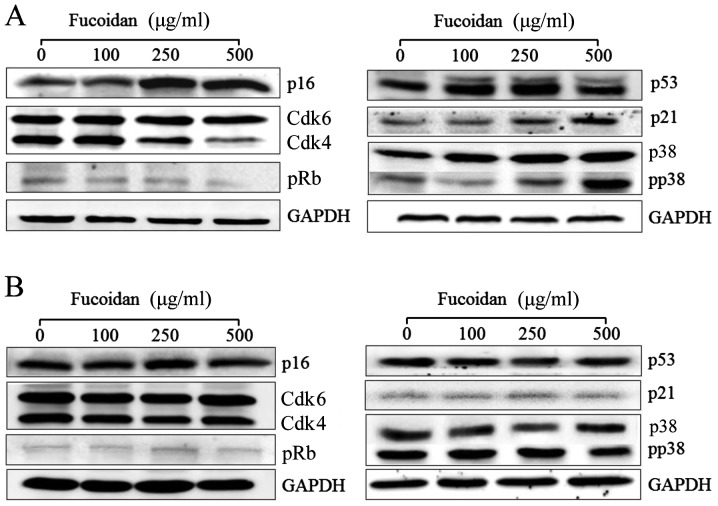
Effects of fucoidan treatment on the p16^INK4a^-pRb pathway and p53, p21, p38 and p-p38 protein expression. GAPDH was used as an internal loading control. HepG2 (A) and Chang-L (B) cells treated with 100, 250 and 500 *μ*g/ml fucoidan for 24 h. Western blotting was performed as described in Materials and methods.

**Figure 5.**
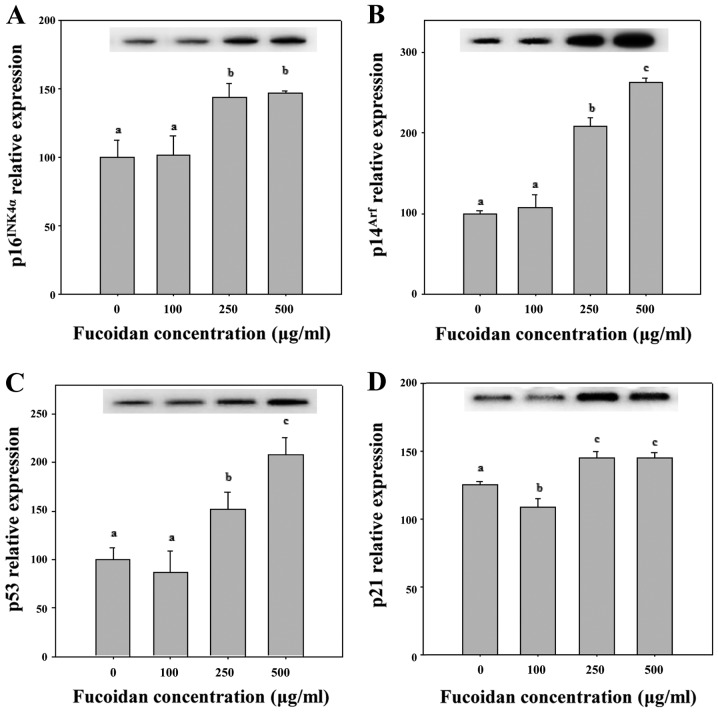
Expression of p16^INK4a^, p14^Arf^, p53 and p21 in HepG2 cells after treatment with fucoidan for 24 h detected by qualitative and real-time PCR. Expression of the GAPDH gene was used as the internal control. Data represent means ± SD of one representative experiment performed in triplicate. Different superscripts represent significantly different results (P<0.05).

The p16^INK4a^ is a key component of the Rb pathway that can inhibit the activity of Cdks, thereby preventing proliferating cells from entering the S phase (22). As shown in Figs. 4A and 5, 250 and 500 *μ*g/ml fucoidan significantly increased p16^INK4a^ expression levels in HepG2 cells (P<0.05). We determined that fucoidan likely activates the Cdk4 and pRb pathway by triggering p16^INK4a^ overexpression and maintaining the hypophosphorylated state of Rb (Figs. 4A and 5). Therefore, we determined that p16^INK4a^ arrests cells in the G1 phase by inhibiting the activities of Cdk4 and pRb (Table II).

**Figure 6.**
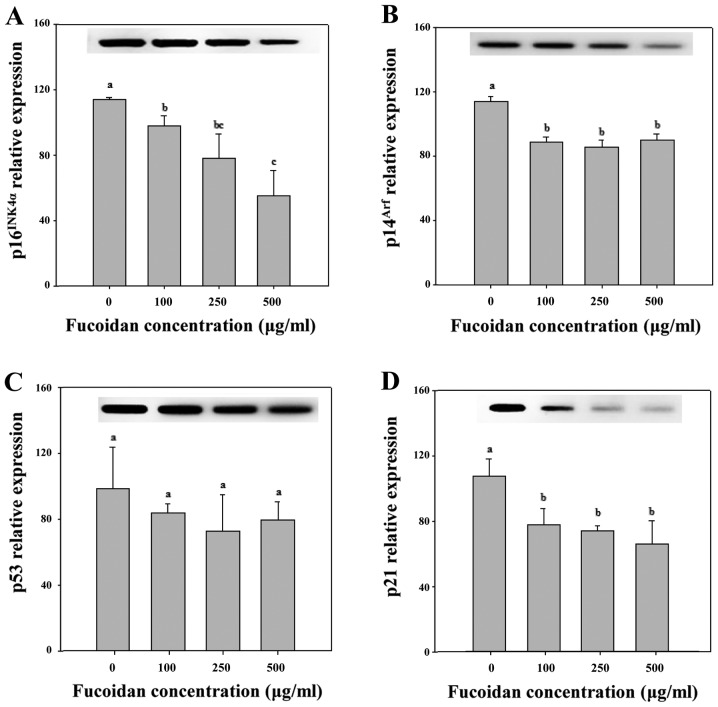
Expression of p16^INK4a^, p14^Arf^, p53 and p21 in Chang-L cells after 24 h treatment with fucoidan as detected by qualitative and real-time PCR. Expression of the GAPDH gene was used as the internal control. Data represent the mean ± SD of one representative experiment performed in triplicate. Different superscripts indicate significantly different values (P<0.05).

To identify the effects of fucoidan that regulate cell growth arrest and thus promote cellular senescence in Chang-L cells, we analyzed p16^INK4a^/Cdk4 and Cdk6/pRb, which are primarily responsible for inhibiting cell growth and inducing cellular senescence (23). The activation of p16^INK4a^ is a common step in the induction of senescence arrest. However, in Chang-L cells treated with fucoidan, overexpression of p16^INK4a^ protein was not detected (Figs. 4B and 6). In addition, Cdk4-dependent activation of pRb, which is important for cell cycle arrest, did not significantly change (Fig. 4). The p16^INK4a^ also links several senescence-initiating signals to p53 activation. However, fucoidan resulted in a significant downregulation of p16^INK4a^ compared to non-treated Chang-L cells when analyzed by real-time PCR (Fig. 6).

### Expression of p14^Arf^-p53 pathway-related proteins in fucoidan-treated cells

The p14^Arf^ and p16^INK4a^ are key tumor suppressor genes that inactivate p53. To explore the effects of the p14^Arf^-p53 pathway on maintaining senescence arrest by fucoidan, we analyzed the expression of the pathway in Chang-L cells by western blotting and real-time PCR. We further investigated the tumor suppressor activity of fucoidan in HepG2 cells through the p14^Arf^-p53 pathway.

Although p14^Arf^ protein expression was not detected by western blotting, the expression of p14^Arf^ mRNA was detected in both HepG2 and Chang-L cells. Real-time PCR determined that fucoidan significantly increased p14^Arf^ mRNA expression in HepG2 cells at concentrations of 250 and 500 *μ*g/ml (Fig. 5) but significantly decreased expression in Chang-L cells (Fig. 6). Hence, fucoidan suppresses p14^Arf^ expression as well as p16^INK4a^ expression in Chang-L cells.

In parallel experiments, treatment of HepG2 cells with 250 and 500 *μ*g/ml fucoidan increased the protein expression of p53 and p21, which are involved in the activation of tumor suppressors (Fig. 4A), and upregulated p53 mRNA. Furthermore, fucoidan-induced p53 mRNA resulted in upregulation of p21 mRNA (Figs. 4A and 5).

In Chang-L cells, the mRNA levels of p14^Arf^ and p21, which are involved in cellular senescence, were significantly lower at concentrations >100 *μ*g/ml (Fig. 6). However, fucoidan treatment did not significantly affect p53 mRNA compared to controls (Fig. 6). Thus, decreased expression of p14^Arf^ and p21 induced senescence arrest due to inactivation of the p53 pathway, and significant changes in the cell cycle were observed after treatment with fucoidan (Fig. 4B, Table II).

### Expression of p38 MAPK in fucoidan-treated cells

**Figure 7.**
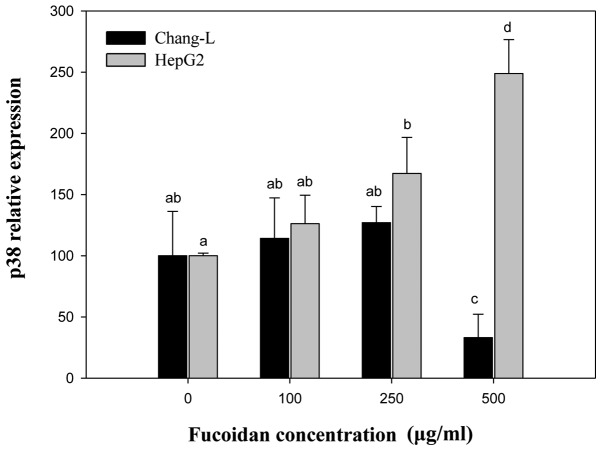
Expression of p38 MAPK mRNA in HepG2 and Chang-L cells after 24 h treatment with fucoidan. Expression of the GAPDH gene was used as the internal control. Data represent the mean ± SD of one representative experiment performed in triplicate. Different superscripts indicate significantly different values (P<0.05).

p38 MAPK can trigger premature senescence in primary cells and permanent oncogene-induced proliferative arrest, which has been proposed as an anti-tumorigenic defense mechanism that induces p53 phosphorylation and upregulation of p16^INK4a^ (18). We defined whether activation of p38 MAPK mediated tumor suppression and replicative senescence, and examined the relationship between fucoidan-stimulated activation of p16^INK4a^/p53 and p38 MAPK in HepG2 and Chang-L cells (Fig. 1). We further determined whether the expression of phosphorylated p38 MAPK increased in both cell types. Fucoidan dose-dependently elevated phospho-p38 MAPK (Fig. 4A) and p38 MAPK gene expression in HepG2 cells (Fig. 7). In contrast, it did not significantly affect p38 and phospho-p38 protein levels in Chang-L cells (Fig. 4B).

Correlation analysis was used to examine the relevance of p38 MAPK, p16^INK4a^ and p53 expression after fucoidan treatment of HepG2 and Chang-L cells. Positive correlations between p38 MAPK and p16^INK4a^/p53 were found in HepG2 cells (Fig. 9), whereas p38 MAPK was closely related with p16^INK4a^ in Chang-L cells (data not shown). However, the levels of p53 mRNA were not associated with p38 MAPK (data not shown).

### Gene expression of α_2_M as an aging biomarker

The expression of α_2_M can easily be measured by real-time PCR and it is a more suitable biomarker candidate of aging then well-known senescence-associated genes such as p16^INK4a^.

**Figure 8.**
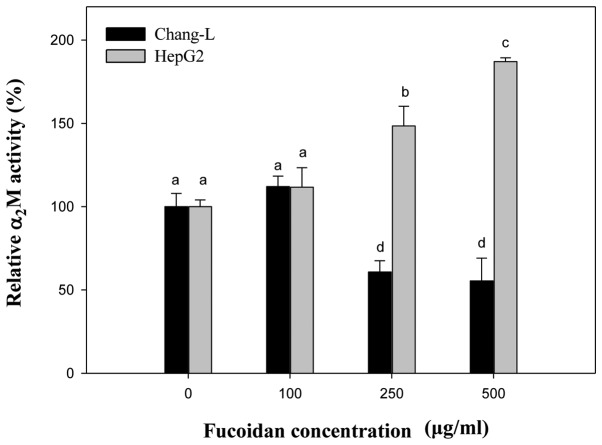
Expression of α_2_M mRNA in HepG2 and Chang-L cells after 24 h treatment with fucoidan. Expression of the GAPDH gene was used as the internal control. Data represent the mean ± SD of one representative experiment performed in triplicate. Different superscripts indicate significantly different values (P<0.05).

**Figure 9.**
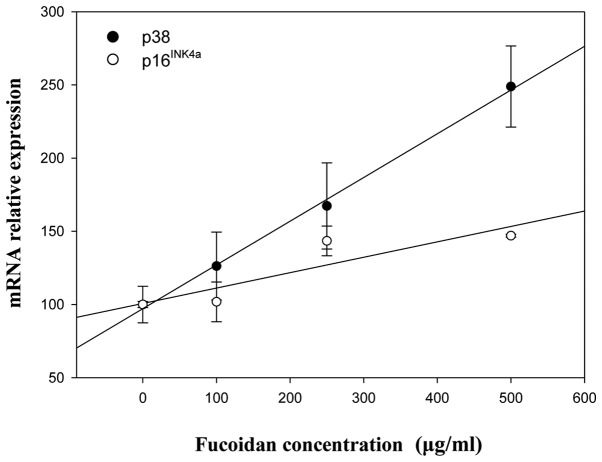
Correlation between p38 MAPK and p16^INK4a^ in HepG2 cells after fucoidan incubation. There was a positive correlation between p38 expression and p16^INK4a^ (r=0.8747, P=0.0987).

Compared to controls, the mRNA level of α_2_M significantly increased in HepG2 cells treated with fucoidan in a manner similar to p16^INK4a^ expression (Fig. 8). In Chang-L cells, fucoidan treatment dose-dependently decreased α_2_M expression, but not p16^INK4a^. When we compared HepG2 and Chang-L cells, a significant difference in α_2_M mRNA levels was noted after incubation with 250 and 500 *μ*g/ml fucoidan (Fig. 8).

## Discussion

Natural products have played a pivotal role in the quest to develop novel chemotherapeutic agents with enhanced specificity and potency in liver cancer. Many marine compounds have chemopreventive and chemotherapeutic effects through various cell-signaling pathways involved in the transduction of mitogenic signals and subsequent regulation of cell growth and proliferation (24).

The diverse biological activities of fucoidan have been studied intensively and include anti-oxidant, immunomodulatory, anti-virus and anti-coagulant effects (3,4,25,26). In particular, the anti-tumor activity has recently attracted considerable attention and several studies have addressed its anti-carcinogenic effects (7). Fucoidan inhibits the growth of a wide variety of tumor cells (3,26), which has become a focus of great interest because it is expected to be a new candidate for low-toxicity cancer therapy (7).

Cancer cells need to evade anti-proliferative signals that negatively regulate growth and proliferation. Cancer cells can avoid this control step by losing the physiological function of the pRb, which controls all anti-proliferative signals (24). Consequently, natural product compounds that inhibit constitutive hyper-phosphorylation of pRb contribute efficiently to the reestablishment of regulated growth in cancer (24). In cancer cells, the hyper-proliferation stress response tends to be suppressed, allowing the continued proliferation of cells carrying overactive mitogenic signals. Hyper-proliferation signals lead to increased levels of p16^INK4a^ and p14^Arf^ resulting in cell cycle arrest or cell death through the pRb and p53 pathways. In many cell types, particularly in humans, the Cdk inhibitor p16^INK4a^ contributes to the cell cycle arrest that occurs after hyper-proliferation stress. Thus, the loss of p16^INK4a^, which occurs in many cancers, helps abolish this response in some cell types. Some cancer-associated chromosomal deletions disrupt both p16^INK4a^ and p14^Arf^ genes, thereby knocking out regulators of both the pRb and p53 pathways. Loss of p53 function is a remarkably common event in tumor cells because it allows cell proliferation to continue following different forms of stress and DNA damage (27). In addition, the *INK4*/*Arf* locus gene, p16^INK4a^ and p14^Arf^ (or p19^Arf^ in mouse), which is upregulated during aging (28) has been genetically linked to numerous aging-associated diseases in humans (29). Accordingly, we anticipated that marine compounds such as fucoidan could have chemopreventive and chemotherapeutic effects by regulating the expression of p16^INK4a^, p14^Arf^ and p53 in cancer cells.

Fedorov *et al* showed that the natural marine chamigrane-type sesquiterpenoid dactylone inhibited cyclin D, Cdk4 expression and pRb phosphorylation (30). The inhibition of these cell cycle components was followed by cell cycle arrest at the G_1_-S transition, with subsequent p53-independent apoptosis in human cancer cells. Park *et al* described the suppression of U937 human monocytic leukemia cell growth by dideoxypetrosynol A, a polyacetylene from the sponge *Petrosia* sp., via induction of the Cdk inhibitor p16^INK4a^ and downregulation of pRb phosphorylation (31).

Interestingly, the wild-type p53 gene is often inactivated in HepG2 cells (32). However, we determined that fucoidan significantly upregulated the expression of p53 in HepG2 cells, while simultaneously inducing apoptosis with inhibition of cellular proliferation (Figs. 2 and 3). Real-time PCR and western blotting studies correlated increased mRNA and protein expression for p16^INK4a^ and p21 (Figs. 4A and 5). These results suggest that the growth arrest in hepatocellular carcinoma cells results from an increase in p53-mediated p21 expression as cells enter senescence, followed by a sustained elevation of p16^INK4a^ (Fig. 5).

The p21 gene is a cell cycle inhibitor and tumor suppressor downstream of p53. When cells are damaged, p53 and p21 act together to inactivate the cyclin-Cdk complex, which could mediate G_1_ and G_2_/M arrest (33). In this study, induction of G_1_ arrest by fucoidan was accompanied by a large accumulation of p53 and p21. In particular, p21 sustains hypophosphorylated Rb and arrests cells in the G_1_ phase. Therefore, we can deduce that the anti-proliferative effect of fucoidan regulates pRb- or p53-mediated cell cycle arrest in HepG2 cells (Table II). This supports the idea that p16^INK4a^-pRb-mediated G_1_ arrest by fucoidan is elicited by p53 and p21 upregulation.

Fujii *et al* suggested that the expression of *INK4a*/*Arf* locus genes p16^INK4a^ and p14^Arf^ could be related to cellular senescence and apoptosis and reported that expression of this locus was increased by valproic acid treatment and induced apoptosis in sphere cells from rat sarcomas (34). We also demonstrated that fucoidan caused induction of apoptosis and tumor suppression by p16^INK4a^ and p14^Arf^ overexpression in HepG2 cells but did not affect normal Chang-L cells. Resistance to apoptosis by cancer cells can be acquired through a variety of strategies, including p53 tumor suppressor gene inactivation. The p53 can lead to induction of the apoptotic cascade (24). However, numerous studies have determined that p38 MAPK may be correlated with apoptosis in various cancer cells (35). The p38 MAPK can regulate cell proliferation and apoptosis through phosphorylation of p53, increased c-myc expression and regulation of Fas/FasL-mediated apoptosis (36). The impact of p38 MAPK on cell cycle regulators plays a crucial role in oncogene-induced senescence involved in the suppression of tumorigenesis and replicative senescence (18). Indeed, we found that fucoidan induced the activation of p38 protein and mRNA in HepG2 cells. Our results show that activation of p38 leads to increased expression of p16^INK4a^, similarly to previous reports on oncogene-induced senescence (19). These studies indicate that p53 is a downstream effector of p38 MAPK, which has been proposed to function as an anti-tumorigenic defense mechanism by inducing p53 and upregulating p16^INK4a^.

We found that fucoidan treatment induced phosphorylation of p38 MAPK and concurrently increased p38 MAPK in HepG2 cells. Consistent with these results, a previous study determined that the anti-tumor activity of fucoidan was mediated by the induction of apoptosis through the activation of p38 MAPK in human colon carcinoma cells (37). Interestingly, in a previous study, honokiol, a novel antitumor agent isolated from a plant, increased the phosphorylated p38 MAPK without affecting p38 expression in HepG2 cells (35). Numerous studies have demonstrated that increased levels of phosphorylated p38 are correlated with malignancy in various cancers (38,39). These data support the hypothesis that fucoidan may have therapeutic potential for cancer treatment.

In summary, in HepG2 cells, it is apparent that p16^INK4a^ upregulation is a key event in anti-tumor activity, and that fucoidan-induced overexpression of p38 MPAK is associated with the p14^Arf^-p53 pathway during apoptosis. This suggests that fucoidan treatment can induce growth-suppressive signals from both p16^INK4a^-Rb and p14^Arf^-p53 pathways, a valuable therapeutic strategy for cancer treatment (Fig. 4A). However, in Chang-L cells, no increase in these pathway-related proteins was noted and no evidence for apoptosis was observed after fucoidan treatment (Fig. 4B). Among the marine compounds, lactone spongistatin induces the degradation of XIAP (40), an anti-apoptotic protein that is overexpressed in chemoresistant cancer cells (41). This compound, similar to our results, does not induce apoptosis in healthy peripheral blood cells (40). Therefore, we suggest that fucoidan could have selective chemotherapeutic effects.

Cellular senescence is an aging mechanism that prevents cell renewal, leading to apoptosis and increased expression of the tumor suppressor gene p16^INK4a^ (42). As proof of the stochastic model, early fibroblasts have low levels of p16^INK4a^ but aging fibroblasts show significantly increased p16^INK4a^ expression (43). The loss or inactivation of p16^INK4a^ is correlated with cell immortality (44). Given the postulated importance of p16^INK4a^ in cell senescence, it is expected that inhibition of p16^INK4a^ would extend the proliferative life span of cultured cells (45). In the present study, we investigated in detail the pathways induced by fucoidan in a normal liver cell line. The expression of p16^INK4a^ and p14^Arf^ mRNA significantly decreased with increasing fucoidan concentration (Fig. 6). Carnero *et al* used a strategy to express antisense p16^INK4a^ and p19^Arf^ (p14^Arf^ in human) RNA in primary mouse embryonic fibroblasts (MEFs) (46). Consequently, the lifespan of MEFs was extended, and a percentage of these cells eventually became immortal. Their study suggested that cellular immortality derived from p16^INK4a^ acts through the Rb pathway, whereas p19^Arf^ acts through both the p53 and Rb pathways. Furthermore, Jung *et al* determined that the p14^Arf^-p53-p21 pathway, in addition to the p16^INK4a^-Rb pathway, controls senescence (47). Phosphorylation of Rb results in increased p16^INK4a^ expression, which inhibits Cdk4/6 resulting in increased levels of hypophosphorylated Rb and decreased p16^INK4a^ expression (12,13). The p14^Arf^-p53 pathway is important as part of the normal life cycle of many cells; it regulates a cell’s entrance into senescence (48). In the present study, the expression of p14^Arf^ and p21 mRNA was significantly downregulated, regardless of protein expression in Chang-L cells. However, the expression of p53 did not appear to change significantly under the same conditions. In agreement, several experimental studies have observed p14^Arf^ in senescent human fibroblasts, independent of p53 (16,48). Moreover, the upregulation of p21 in aging and senescent human fibroblasts is well documented (11,49).

It should be noted that *INK4a/Arf* expression is both a biomarker and effector of aging. Our results suggest that fucoidan prevented senescence in hepatoma cells by mediating a process that included a decrease in p14^Arf^ expression as cells entered quiescence followed by a decline in the level of p16^INK4a^. We must be clear that these results do not negate an anti-aging effect of fucoidan in normal hepatic cells, but only indicate that reduced levels of these proteins are not enough to cause cellular longevity. Therefore, we examined other clinical biomarkers of aging. Like oncogene-induced senescence, replicative senescence is identified by senescence biomarkers such as SA-β-gal and α_2_M. The α_2_M and SA-β-gal expression is accompanied by increased expression of negative growth regulators including p53, p21, p16^INK4a^ and p14^Arf^ (20). α_2_M is a major plasma protein that functions as a panprotease inhibitor. Ma *et al* showed that expression of p16^INK4a^ at each passage was exponentially correlated with cumulative population doubling level (PDL) (21), in agreement with previous reports on p16^INK4a^ (45), and provided further evidence that mRNA expression of α_2_M had a positive linear correlation with cumulative PDL, similar to p16^INK4a^. These results, including the positive relationship between α_2_M and p16^INK4a^, suggest that α_2_M mRNA expression can be used as a biomarker of cellular senescence (21). Another study found that the amount of α_2_M fragment derived from culture medium increased as the cells aged (50). We attempted to identify changes that are essential for a cellular anti-aging response by comparing the effects of fucoidan on α_2_M mRNA expression in HepG2 and Chang-L cells. We found that expression of α_2_M was upregulated in HepG2 cells, but significantly downregulated in Chang-L cells after incubation with fucoidan. These results suggest that fucoidan has the anti-senescence effect in normal hepatic cell line. As we have seen, there are several independent pathways that control replicative senescence in human cells.

In conclusion, fucoidan arrests cells in the G_1_ phase through p16^INK4a^ and p14^Arf^ in HepG2 cells. It also increases the activity of tumor suppressor proteins p53 and p38 MAPK, which play a critical role in the regulation of apoptosis. Accordingly, in addition to directly inhibiting the proliferation of tumor cells, fucoidan may also restrain the development of tumor cells by inducing apoptosis. Fucoidan also affected the senescence of Chang-L cells by decreasing mRNA expression of p16^INK4a^, p14^Arf^, p21 and the senescence biomarker α_2_M. These findings suggest that fucoidan may offer substantial therapeutic potential for cancer treatment without inducing senescence in normal cells, and that it may be possible to use fucoidan therapeutically.

## Figures and Tables

**Table I.** Primers for real-time PCR.

	Forward	Reverse
GAPDH	TGC ACC ACC AAC TGC TTA GC	GGC ATG GAC TGT GGT CAT GAG
p16^INK4a^	GCC CAA CGC CCC GAA CTC TTT C	TGA AGC TAT GCC CGT CGG TCT G
p14^Arf^	GGA GGC GGC GAG AAC AT	TGA ACC ACG AAA ACC CTC ACT
p53	GAG GGA TGT TTG GGA GAT GTA A	CCC TGG TTA GTA CGG TGA AGT G
p38	GAG GAT GCC AAG CCA TG	TCT TAT CTG AGT CCA ATA CAA GCA TC
p21	CCG CGA CTG TGA TGC GCT AAT G	CTC GGT GAC AAA GTC GAA GTT C
α_2_M	TTG TCA GTG ACG TTT GCC TC	CAA ACT CAT CCG TCT CGT AG

**Table II.** Fractions of each cell cycle phase in HepG2 and Chang-L cells cultured in the presence of fucoidan for 24 h.

Fucoidan in HepG2(*μ*g/ml)	G_0_/G_1_ (%)	S (%)	G_2_/M (%)

0	43.12±6.50^a^	28.86±4.20^a^	23.86±5.91^a^
100	68.52±4.67^b^	21.74±5.98^a,b^	8.26±5.33^b^
250	71.76±2.37^b,c^	17.60±3.03^b^	13.90±1.25^b^
500	77.78±2.00^c^	8.94±1.38^c^	12.20±2.23^b^

Fucoidan in Chang-L(*μ*g/ml)	G_0_/G_1_ (%)	S (%)	G_2_/M (%)

0	44.18±2.12^a^	23.68±2.06^a^	30.78±2.65^a^
100	45.67±1.05^a^	18.67±1.47^b^	35.67±1.07^b^
250	43.83±1.60^a^	25.63±1.51^a^	29.30±2.82^a^
500	45.50±5.24^a^	25.63±1.51^a^	28.00±5.91^a^

Data represent means ± SD, n=5. Different superscripts represent significantly different results (P<0.05).

## References

[1] Li Y, Nichols MA, Shay JW, Xiong Y (1994). Transcriptional repression of the D-type cyclin-dependent kinase inhibitor p16 by the retinoblastoma susceptibility gene product pRb. Cancer Res.

[2] Dürig J, Vruhn T, Zurborn KH, Gutensohn K, Bruhn HD, Bèress L (1997). Anticoagulant fucoidan fractions from *Fucus vesiculosus* induce platelet activation *in vitro*. Thromb Res.

[3] Maruyama H, Tamauchi H, Hasimoto M, Nakano T (2003). Antitumor activity and immune response of Mekabu fucoidan extracted from Sporophyll of *Undaria pinnatifida*. In vivo.

[4] Wang J, Zhang Q, Zang Z, Li Z (2008). Antioxidant activity of sulfated polysaccharide fractions extracted from *Laminaria japonica*. Int J Biol Macromol.

[5] Hu T, Liu D, Chen Y, Wu J, Wang S (2010). Antioxidant activity of sulfated polysaccharide fractions extracted from *Undaria pinnitafida in vitro*. Int J Biol Macromol.

[6] Lee H, Kim JS, Kim E (2012). Fucoidan form seaweed *Fucus vesiculosus* inhibits migration and invasion of human lung cancer cells via PI3K-Akt-mTOR pathways. PLoS One.

[7] Xue M, Ge Y, Zhang J (2012). Anticancer properties and mechanisms of fucoidan on mouse breast cancer in vitro and in vivo. PLoS One.

[8] Kim NY, Sun JM, Kim YJ (2010). Cisplatin-based combination chemotherapy for advanced hepatocellular carcinoma: a single centre experience before the sorafenib era. Cancer Res Treat.

[9] Rayess H, Wang MB, Srivatsan ES (2012). Cellular senescence and tumor suppressor gene p16. Int J Cancer.

[10] Krishnamurthy J, Torrice C, Ramsey MR (2004). Ink4a/Arf expression is a biomarker of aging. J Clin Invest.

[11] Stein GH, Drullinger LF, Soulard A, Dulić V (1999). Differential roles for cyclin-dependent kinase inhibitors p21 and p16 in the mechanisms of senescence and differentiation in human fibro-blasts. Mol Cell Biol.

[12] Li B, Lu F, Wei X, Zhao R (2008). Fucoidan: structure and bioactivity. Molecules.

[13] Hara E, Smith R, Parry D, Tahara H, Stone S, Peters G (2008). Regulation of p16CDKN2 expression and its implications for cell immortalization and senescence. Mol Cell Biol.

[14] Haber DA (1997). Splicing into senescence: the curious case of p16 and p19^Arf^. Cell.

[15] Pomerantz J, Schreiber-Agus N, Liégeois NJ (1998). Ink4a tumor suppressor gene product, p19^Arf^, interacts with MDM2 and neutralizes MDM2’s inhibition of p53. Cell.

[16] Dimri GP, Itahana K, Acosta M, Campisi J (2000). Regulation of a senescence checkpoint response by the E2F1 transcription factor and p14^Arf^tumor suppressor. Mol Cell Biol.

[17] Sharpless NE (2005). INK4a/ARF: A multifunctional tumor suppressor locus. Mutat Res.

[18] Han J, Sun P (2007). 2007. The pathways to tumor suppression via route p38. Trends Biochem Sci.

[19] Bulavin DV, Fornace AJ (2004). p38 MAPK kinase’s emerging role as a tumor suppressor. Adv Cancer Res.

[20] Serrano M, Lin AW, McCurrach ME, Beach D, Lowe SW (1997). Oncogenic ras provokes premature cell senescence associated with accumulation of p53 and p16^INK4a^. Cell.

[21] Ma H, Li R, Zhang Z, Tong T (2004). mRNA level of alpha-2-macro-globulin as an aging biomarker of human fibroblasts in culture. Exp Gerontol.

[22] Serrano M (2000). The INK4a/ARF locus in murine tumorigenesis. Carcinogenesis.

[23] Schmitt CA, Fridman JS, Yang M (2002). A senescence program controlled by p53 and p16^INK4a^contributes to the outcome of cancer therapy. Cell.

[24] Schumacher M, Kelkel M, Dicato M, Diederich M (2011). Gold from the sea: marine compounds as inhibitors of the hallmarks of cancer. Biotechnol Adv.

[25] Ishii H, Iwatsuki M, Ieta K (2008). Cancer stem cells and chemoradiation resistance. Cancer Sci.

[26] Alekseyenko TV, Zhanayeva SY, Venediktova AA (2007). Antitumor and antimetastatic activity of fucoidan, a sulfated polysaccharide isolated from the Okhotsk Sea *Fucus evanescens* brown alga. Bull Exp Biol Med.

[27] Meek DW (2009). 2009. Tumour suppression by p53: a role for the DNA damage response?. Nat Rev Cancer.

[28] Ressler S, Bartkova J, Niederegger H (2006). p16^INK4a^is a robust in vivo biomarker of cellular aging in human skin. Aging Cell.

[29] Melzer D (2008). Genetic polymorphisms and human aging: association studies deliver. Rejuvenation Res.

[30] Fedorov SN, Shubina LK, Bode AM, Stonik VA, Dong Z (2007). Dactylone inhibits epidermal growth factor-induced transformation and phenotype expression of human cancer cells and induces G1-S arrest and apoptosis. Cancer Res.

[31] Park C, Kim GY, Kim GD (2006). Suppression of U937 human monocytic leukemia cell growth by dideoxypetrosynol A, a polyacetylene from the sponge Petrosia sp., via induction of Cdk inhibitor p16 and down-regulation of pRB phosphorylation. Oncol Rep.

[32] Müller M, Strand S, Hug H (1997). Drug-induced apoptosis in hepatoma cells is mediated by the CD95 (APO-1/Fas) receptor/ligand system and involves activation of wild-type p53. J Clin Invest.

[33] Vogelstein B, Lane D, Levine AJ (2000). Surfing the p53 network. Nature.

[34] Fujii H, Honoki K, Tsujiuchi T (2007). Reduced expression of INK4a/ARF genes in stem-like sphere cells from rat sarcomas. Biochem Biophysic Res Commun.

[35] Deng J, Qian Y, Geng L (2008). Involvement of p38 mitogen-activated protein kinase pathway in honokiol-induced apoptosis in a human hepatoma cell line (HepG2). Liver Int.

[36] Wang YX, Xu XY, Su WL (2009). Activation and clinical significance of p38 MAPK signaling pathway in patients with severe trauma. J Surg Res.

[37] Hyun JH, Kim SC, Kang JI (2009). Apoptosis inducing activity of fucoidan in HCT-15 colon carcinoma cells. Biol Pharm Bull.

[38] Iyoda K, Sasaki Y, Horimoto M (2003). Involvement of the p38 mitogen-activated protein kinase cascade in hepatocellular carcinoma. Cancer.

[39] Wagner EF, Nebreda AR (2009). Signal integration by JNK and p38 MAPK pathways in cancer development. Nat Rev Cancer.

[40] Schyschka L, Rudy A, Jeremias I, Barth N, Pettit GR, Vollmar AM (2008). Spongistatin 1: a new chemosensitizing marine compound that degrades XIAP. Leukemia.

[41] Igney FH, Krammer PH (2002). Death and anti-death: tumor resistance to apoptosis. Nat Rev Cancer.

[42] Verzola D, Gandolfo MT, Gaetani G (2008). Accelerated senescence in the kidneys of patients with type 2 diabetic nephropathy. Am J Physiol Renal Physiol.

[43] Tsygankow D, Liu Y, Sanoff HK, Sharpless NE, Elston TC (2009). A quantitative model for age-dependent expression of the p16^INK4a^tumor suppressor. Proc Natl Acad Sci USA.

[44] Huschtscha LI, Reddel RR (1999). p16^INK4a^and the control of cellular proliferative life span. Carcinogenesis.

[45] Duan J, Zhang Z, Tong T (2001). Senescence delay of human diploid fibroblast induced by anti-sense p16^INK4a^expression. J Biol Chem.

[46] Carnero A, Hudson JD, Price CM, Beach DH (2000). p16^INK4A^and p19ARF act in overlapping pathways in cellular immortalization. Nat Cell Biol.

[47] Jung YS, Qian Y, Chen X (2010). Examination of the expanding pathway for the regulation of p21 expression and activity. Cell Signal.

[48] Collado M, Blasco MA, Serrano M (2007). Cellular senescence in cancer and aging. Cell.

[49] Tahara H, Sato E, Noda A, Ide T (1995). Increase in the expression level of p21*sdi1*/*cip1*/*waf1* with increasing division age in both normal and SV40-transformed human fibroblasts. Oncogene.

[50] Kondo T, Sakaguchi M, Namba M (2001). Two-dimensional gel electrophoresis studies on the cellular aging: accumulation of alpha-2-macroglobulin in human fibroblasts with aging. Exp Gerontol.

